# T cell immune memory after covid-19 and vaccination

**DOI:** 10.1136/bmjmed-2022-000468

**Published:** 2023-11-22

**Authors:** Lulu Wang, Alex Nicols, Lance Turtle, Alex Richter, Christopher JA Duncan, Susanna J Dunachie, Paul Klenerman, Rebecca P Payne

**Affiliations:** 1 Translational and Clinical Research Institute, Immunity and Inflammation Theme, Newcastle University, Newcastle upon Tyne, UK; 2 NIHR Health Protection Research Unit in Emerging and Zoonotic Infections, Institute of Infection, Veterinary and Ecological Sciences, University of Liverpool, Liverpool, UK; 3 Tropical and Infectious Disease Unit, Liverpool University Hospitals NHS Foundation Trust, Liverpool, UK; 4 Institute of Immunology and Immunotherapy, College of Medical and Dental Science, University of Birmingham, Birmingham, UK; 5 Department of Infection and Tropical Medicine, Newcastle Upon Tyne Hospitals NHS Foundation Trust, Newcastle Upon Tyne, UK; 6 NDM Centre For Global Health Research, Nuffield Department of Clinical Medicine, University of Oxford, Oxford, UK; 7 Mahidol-Oxford Tropical Medicine Research Unit, Mahidol University Faculty of Science, Bangkok, Thailand; 8 Oxford University Hospitals NHS Foundation Trust, Oxford NIHR Biomedical Research Centre, University of Oxford, Oxford, Oxfordshire, UK; 9 Translational Gastroenterology Unit, University of Oxford, Oxford, UK

**Keywords:** COVID-19, Immunology, Covid-19, Respiratory tract infections

## Abstract

The T cell memory response is a crucial component of adaptive immunity responsible for limiting or preventing viral reinfection. T cell memory after infection with the SARS-CoV-2 virus or vaccination is broad, and spans multiple viral proteins and epitopes, about 20 in each individual. So far the T cell memory response is long lasting and provides a high level of cross reactivity and hence resistance to viral escape by variants of the SARS-CoV-2 virus, such as the omicron variant. All current vaccine regimens tested produce robust T cell memory responses, and heterologous regimens will probably enhance protective responses through increased breadth. T cell memory could have a major role in protecting against severe covid-19 disease through rapid viral clearance and early presentation of epitopes, and the presence of cross reactive T cells might enhance this protection. T cell memory is likely to provide ongoing protection against admission to hospital and death, and the development of a pan-coronovirus vaccine might future proof against new pandemic strains.

## Introduction

The emergence of the SARS-CoV-2 virus in 2019 caused a pandemic of unprecedented scale, with more than 760 million infections reported worldwide and 6.8 million deaths attributed to covid-19 (15 March 2023). In just over a year, many effective and protective vaccines were developed and administered globally, successfully reducing rates of admission to hospital and death.

Belonging to the family Coronaviridae, SARS-CoV-2 is a positive strand single sense RNA virus with a large genome (~30 kb). Other coronaviruses include the seasonal human coronaviruses, which cause mild common colds, and SARS-CoV-1 and MERS-CoV, which cause severe acute respiratory syndrome and Middle East respiratory syndrome, respectively, both of which also cause severe pneumonia.^1^ T cell memory has a crucial role in defence against viral infections, and immunological studies of other coronavirus infections provide useful insights into the potential long term protective effect of T cell memory in SARS-CoV-2 infection.^2–5^ Historically, human coronaviruses have been described as typically inducing weak T cell immunity and antibody responses that are not well maintained, with reinfections common within 12 months.^2^ Other reports, however, have described long lived antibody and T cell immunity against human coronaviruses, offering protection from symptomatic disease rather than reinfection.^3 5^ Also, SARS-CoV-1 specific T cell responses are maintained for up to 17 years^4^ and MERS-CoV induces T cell induction without seroconversion, both of which indicate that T cells might have a protective role.

The development and roll-out of efficacious vaccines, and the emergence of new SARS-CoV-2 variants, altered the course of the pandemic. Reinfections are now relatively common,^6 7^ the phenotype of the disease is milder,^8^ and hospital admissions are low.^9^ Most of people worldwide are no longer immunologically naive to SARS-CoV-2, either through infection or vaccination or a combination of both (hybrid immunity).^10^ T cell memory has a role in immunity to SARS-CoV-2, induced through infection or vaccination, but an absence of long term sterilising immunity from either raises concern that current immunity to SARS-CoV-2 will fail, leading to a resurgence of infections and hospital admissions. Understanding the development of the T cell response to the SARS-CoV-2 virus, the nature of long term memory, and how this response translates into observed clinical protection, particularly for clinically vulnerable groups, is important for informing ongoing strategies to limit current SARS-CoV-2 infections. These strategies will also help to develop more effective and protective vaccines that can future proof against covid-19 disease. In this review, our aim was to integrate published studies on T cell immune memory to SARS-CoV-2, focusing on the differences between natural, vaccine, and hybrid induced immunity, and to define immune correlates and establish how they might be harnessed for future pandemic preparedness.

## Epidemiology

SARS-CoV-2 infection and reinfection worldwide has had a wide ranging prevalence. Data from 52 studies between 2019 and 2022 estimated that the prevalence of reinfection was 0.3-7.5%, depending on the country of origin.^11^ The number of patients with confirmed SARS-CoV-2 infection was 761 767 759, with 6 784 181 confirmed deaths from covid-19, as of 20 September 2023.^12^ The incidence of SARS-CoV-2 infection was estimated as 20 per 10 000 population weeks during the delta variant dominant period of SARS-CoV-2 infection, 40 per 10 000 population weeks during the first omicron period, and 17 per 10 000 population weeks during the second omicron period.^13^

## Sources and selection criteria

Web of Science was used to search for related articles published between 1 January 2020 and 19 April 2023. Search keywords included results for 2020-23 (year published) AND (covid or sars?cov?2) (topic) AND (vaccination or vaccine or infection or infected or “human*NEAR/5challenge*”) (topic) AND (“T-cell*” or “T cell*” or “immune*”) (topic) AND memory (topic). Truncation symbols were included in the search; * allows the search to find any number of characters including zero, and ? finds one character only and can be repeated. We also used the PubMed database and manual searching for relevant data, including www.who.int and www.ourworldindata.org, and preprint databases, such as medRxiv and bioRxiv. We prioritised high quality, large cohort studies, and excluded small case studies and studies not published in English.

### Covid-19 and emerging variants

**Figure 1:**
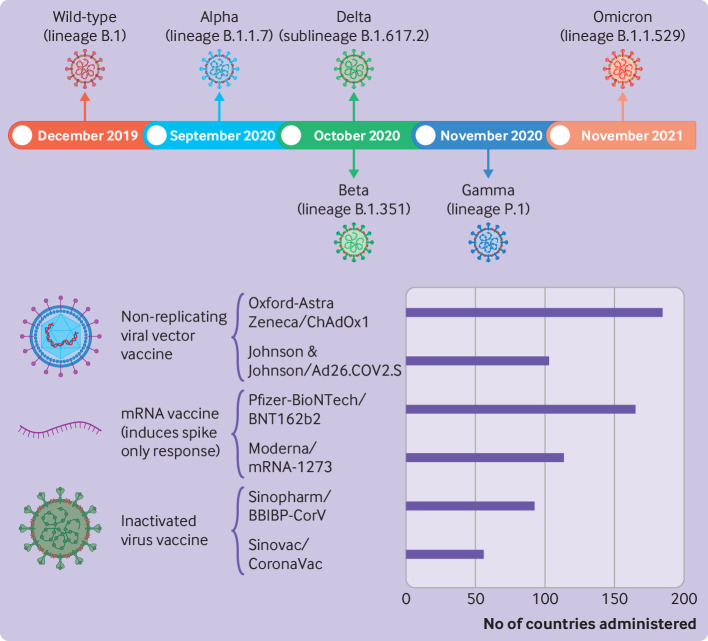
SARS-CoV-2 wild type and variants of concern, and most widely used covid-19 vaccines globally. (Top) Timeline of when the wild type and variants of concern were first detected. (Bottom) Summary of number of countries that the most widely used covid-19 vaccines were administered, and vaccine types. Source of data: https://www.who.int/. Created with BioRender.com

The wild type pandemic strain that emerged in Wuhan, China, towards the end of 2019 and spread worldwide was called B.1, Wuhan-Hu-1, or wild type strain. Although a substantial proportion of people infected with Wuhan-Hu-1 had an asymptomatic SARS-CoV-2 infection (positive polymerase chain reaction (PCR) test result),^14^ covid-19 clinically manifests as a respiratory disease with variable outcomes, ranging from mild, self-limiting symptoms to death. Risk factors associated with severe disease and death include older age, male sex, ethnic group, and comorbidities (diabetes, hypertension, lung disease, malignancy, and immune deficiency).^15 16^ As the pandemic progressed, variants of the SARS-CoV-2 virus emerged (figure 1). The World Health Organization began tracking variants of concern, which are identified genetically because of their potential for biological effects, for increased disease burden, or to evade natural or vaccine induced immunity (distinct from variants of interest or variants under monitoring). Variants of concern have been responsible for further periods of infection worldwide.

By February 2023, WHO had historically declared five variants of concerns. The alpha (B.1.1.7 lineage), beta (B.1.351 lineage), and gamma (P.1 lineage) variants were first identified and declared variants of concerns in late 2020, the delta variant B.1.617.2 lineage was first detected in October 2020 and declared a variant of concern in May 2021, and the omicron B.1.1.529 lineage in November 2021.^17^ Alpha, beta, gamma, and delta variants increased disease burden compared with the wild type strain to varying degrees, but have since been de-escalated as variants of concerns by WHO.^17 18^ By February 2022, omicron viruses accounted for 98% of publicly available sequences.^19^ Omicron causes a milder infection than other variants, but has more than 30 spike protein mutations and extensive escape abilities from both natural and vaccine induced immunity.^7 20 21^ Sublineages of omicron B.1.1.529, which include BA.2, BA.4, and BA.5, were under surveillance by WHO until March 2023,^18^ but have also since been de-escalated as variants of concern because these parental lineages are no longer circulating. As of 17 August 2023, no circulating variants of concern exist, but various descendants of omicron BA.2 and BA.5 are under surveillance as variants of interest (XBB.1.5, XBB.1.16, and EG.5) and variants under monitoring (BA.2.75, CH.1.1, XBB, XBB.1.9.1, XBB.1.9.2, XBB.2.3, and BA.2.86).^22^

### Covid-19 vaccines

The development of covid-19 vaccines became a global priority because of the urgency of the covid-19 pandemic. Current covid-19 vaccine platforms are wide ranging and include traditional protein subunit vaccines, virus-like particles, and inactivated whole virus vaccines, as well as newly developed non-replicating viral vectored and mRNA based vaccine platforms.^23^ As of 2 December 2022, 50 SARS-CoV-2 vaccines had been approved by at least one country, 201 countries had approved vaccines, 242 vaccine candidates were being investigated, with 821 ongoing or completed vaccine trials (https://covid19.trackvaccines.org/).

More than 13 billion vaccine doses have been administered worldwide, reaching 5.5 billion people, estimated at 72% of the world's population.^24^ The most widely used vaccines are Oxford-AstraZeneca's non-replicating viral vector ChAdOx1 (Vaxzevria, 185 countries), Pfizer-BioNTech's mRNA vaccine BNT162b2 (Comirnaty, 165 countries), Moderna's mRNA vaccine mRNA-1273 (Spikevax, 114 countries), Johnson & Johnson's non-replicating viral vector Ad26.COV2.S (Jcovden, 103 countries), Sinopharm's inactivated virus vaccine BBIBP-CorV (Covilo, 93 countries), and Sinovac's inactivated virus vaccine CoronaVac (56 countries) (figure 1). Detailed comparisons of clinical efficacy between the vaccines is difficult because of the differences in clinical set-up between vaccine trials, including population demographic, study size, variations in circulating viral strains, and efficacy reporting. All approved vaccines, however, provide a high level of protection (>90%) from hospital admission and death.^25^ WHO guidelines state that a successful covid-19 vaccine should have >50% efficacy against infection, hospital admission, or death, and therefore the covid-19 vaccination strategies were considered unequivocally successful at controlling infection rates at the point of the vaccine roll-out. A systematic review of 68 clinically controlled or real world observational studies on the long term effectiveness of the most widely used vaccines indicated that vaccine effectiveness from infection wanes over time, reducing to about 60% at five months, but that protection from hospital admission and death remains high at 79% and 86%, respectively, six months after vaccination.^25^

Since September 2022, Pfizer-BioNTech and Moderna have released updated bivalent versions of their vaccines that include the spike sequence from BA.1 as a booster dose.^26 27^ In a recent clinical observational large cohort study of 6.2 million people, 292 659 and 1 070 136 people received monovalent and bivalent boosters, respectively. Researchers found that bivalent boosters were about 60% effective against omicron infection and hospital admission compared with 25% for monovalent boosters, in individuals who had already received 2-4 monovalent vaccine doses.^28^ About 2.7 billion boosters have been administered worldwide, amid concerns about waning protection from covid-19 and the emergence of the omicron variant and sublineages. A systematic review of 68 studies showed that the long term effectiveness of bivalent booster vaccines was reduced from 70% to 43% for infections and from 89% to 71% for hospital admission at 112 days or later.^25^ Collectively, these data indicate that vaccine effectiveness against the omicron strain is marginally adequate and that vaccines provide reasonably stable protection against hospital admission and death in the long term, but protection from infection is modest and wanes over time. Waning protection from SARS-CoV-2 infection might be a result of waning of the immune response, viral escape, or a combination of both.

### T cells and T cell memory

T cell responses have become an important focus in understanding long term protection from covid-19. Antibodies against the SARS-CoV-2 virus wane more rapidly than T cell immunity, and show less cross protection against variants,^29–32^ suggesting that T cells are a major contributing factor in the ongoing protection against hospital admission and death.

T cells are a highly specialised immune cell integral to the adaptive immune response. T cells express a T cell receptor that recognises an antigen on major histocompatibility complex (MHC) molecules expressed by most human cells in the body. VDJ (variability, diversity, and joining) gene rearrangement during thymic development creates more than 100 million unique T cell receptor sequences and hence a highly diverse T cell receptor repertoire for antigen recognition. In humans, these T cell receptors typically recognise short peptides derived from pathogens expressed on MHC class I (CD8+ T cells) or MHC class II (CD4+ T cells) molecules. Other unconventional T cells exist, including the gamma-delta T cell subsets and MAIT cells (mucosal associated invariant T cells), which fall outside the scope of this review.

T cells develop within the thymus and can mature broadly as helper CD4+ T cells and cytotoxic CD8+ T cells. Cytotoxic CD8+ T cells can recognise and kill virally infected cells, thus providing local control of viral infection in tissues. Helper CD4+ T cells provide help, especially to B cells, required for development of a mature antibody response. Thus both sets of T cells are potentially involved in optimal viral defence and protection in different ways, even though they might be measured together in some assays (table 1). To protect against infection and disease, CD8+ T cells need to recognise an infected cell, and therefore in principle, CD8+ T cells cannot provide completely sterile protection. CD8+ T cells can ensure rapid clearance of virus, however, and many examples exist from other infections where CD8+ T cells have been associated with protection, including respiratory infections, such as influenza.^33^

**Table 1:** Techniques to assess T cell immunity

Assay	Overview	Strengths	Limitations
ELISpot	In its simplest form, this assay measures one cytokine from bulk T cells, detecting the secretion of cytokine (usually interferon γ) trapped by a pre-coated antibody on a polyvinylidene difluoride or nitrocellulose membrane. Cytokine secreting cells are visualised as spots by an enzyme coupled secondary antibody. Read-out is magnitude of cytokine secreting cells as a proportion of bulk cells	Highly sensitive Can be standardised Not labour intensive Easy to scale up	Limited in the number of cytokines Simple read-out and lack of phenotypic information Biased towards effector cells Requires stimulation with exogenous peptide Does not distinguish between CD4+ and CD8+ T cells
Intracellular cytokine staining	This assay measures the production and accumulation of cytokines within the endoplasmic reticulum after in vitro stimulation of the T cell receptor with exogenous peptide, which triggers the T cell receptor in an antigen specific way. Fluorescently labelled antibodies and flow cytometry are used to detect positive cytokine responding antigen specific cells	Quantitate and qualitative read-out Can be combined with other flow cytometry protocols (eg, cell surface markers or MHC multimers, or both) to phenotype the responding cells	Less sensitive than ELISpot Relies on previous knowledge and thus can be biased towards detection of a particular subset of T cell Labour intensive and difficult to scale up Requires stimulation using exogenous peptide
MHC tetramer (multimer)	Fluorescent labelled complex of multiple MHC class I or class II glycoproteins with loaded peptide that can bind the T cell receptor on T cells, allowing identification of these cells with flow cytometry	No stimulation of cells required Subset and phenotype of responding cells HLA peptide tetramers are a reagent that can stain antigen specific T cells without the need for activation by preincubation with peptides	Require cloned MHC molecules Need previous knowledge of HLA type for multimer preparation HLA class I reagents more well established than HLA class II reagents, hence bias towards CD8+ T cell responses Labour intensive and difficult to scale up Multimers often do not cover the whole T cell repertoire HLA bias caused by reagent availability
Activation induced marker	Detect antigen specific T cells based on upregulation of T cell receptor stimulated surface markers. Requires non-physiological cell stimulation with exogenous peptide	Do not rely on previous knowledge of the epitope or HLA type Not limited by predetermination of cytokines Can determine subset and phenotype of responding cells	Limited information on functional activity Inability to distinguish between different T cell subsets because of bystander activation Labour intensive and difficult to scale up
Proliferation	Assay assesses T cell proliferation in response to peptide stimulation in vitro over period of about 6 days. Typically cellular proliferation is measured by flow cytometric assessment of dilution of a fluorescent dye loaded into the T cells at rest	Highly sensitive Can detect resting memory cells below that seen with cytokine release assays, such as ELISpot or intracellular cytokine staining Does not rely on specific cellular function such as interferon γ Can define CD4+ and CD8+ subsets	Quantitation not standardised across assays No data on effector function of cells Labour intensive
Cytokine release assay	Assay assesses cellular cytokine release in response to peptide stimulation in whole blood or peripheral blood mononuclear cells. Cytokine is measured by ELISA or proximity qPCR	Highly sensitive Functionality of cells Can be standardised although multiple cytokine release assay methodologies exist Easy to scale up and high throughput Can be multiplexed Not labour intensive	Quantitation not necessarily standardised across assays Inability to distinguish between different T cell subsets Multiplexing might be difficult to optimise because of different conditions required for cytokine release Requires exogenous peptide

ELISpot=enzyme linked immunosorbent spot; ELISA=enzyme linked immunosorbent assay; qPCR=quantitative polymerase chain reaction; MHC=major histocompatibility complex.

The assessment of overall virus specific T cell responses, and subsequent identification of correlates of protection, is more challenging than with humoral immunity for a number of reasons. Diversity exists for the T cell receptor repertoire and MHC genes, and hence the breadth and diversity of any individual's viral antigen specific T cell response. Also, T cells at immunologically relevant sites, such as mucosal tissue, tend to be difficult to access, and therefore the use of circulating peripheral blood T cells as a proxy is common in larger studies. Peripheral blood is minimally invasive to access, less distressing for patients because blood sampling often coincides with routine clinical blood tests, and can provide access to T cells in sufficient quantities for high throughput assays. T cell assays typically require some skilled processing after blood samples are obtained to isolate the cells or interest, however, and the cryopreservation and subsequent thawing of live cells adds to the complexity in evaluating and standardising T cell immunity.

Techniques to assess antiviral T cells include interferon γ enzyme linked immunospot (ELISpot) assay, activation induced marker assay,^34 35^ intracellular cytokine staining, cytokine release assays,^36^ HLA peptide multimer based assays, and proliferation assays. Other cytokine release assays have also been tested for the SARS-CoV-2 virus to increase scalability.^37^ All of these assays measure antigen specific T cells within a complex mixture of cells, such as peripheral blood mononuclear cells (table 1).

### T cell memory after SARS-CoV-2 infection

Systemic SARS-CoV-2 specific T cells can be detected in patients who have recovered from covid-19, as well as in those who were infected but had no symptoms, with individual differences in the trajectory of waning of the T cell response over time.^38^ After recovery from infection, memory SARS-CoV-2 specific T cell responses have a half-life of about 200 days, and have been shown to be maintained for up to one year, with CD4+ and CD8+ T cells comprising 0.5% and 0.8% of the T cell repertoire, targeting about 19 and 17 epitopes, respectively.^39 40^ These studies are limited to the most dominant proteins targeted by the most common HLA types, however, and not all HLA restrictions have been experimentally confirmed. Furthermore, differences in ethnic group, sex, and age have not been fully explored. The SARS-CoV-2 genome encodes 29 proteins, many of which encode T cell epitopes, including the out-of-frame open reading frames. More than 1400 potential epitopes have been identified so far, and patterns of immunodominance are emerging, including public epitopes shared between individuals.^36 41–43^

The phenotype of the T cell memory response is critical for its effectiveness on re-exposure, and this response has been the focus of longitudinal cohort studies, which include strategies that use high dimensional single cell transcriptional analysis as well as proteomic and functional assays. SARS-CoV-2 specific CD8+ memory T cells, assessed one year after infection, have a long lived immune signature expressing CD45RA, interleukin 7 receptor, and T cell factor 1, but low levels of C-C chemokine receptor type 7 (CCR7), resembling the phenotype of long lived effector memory T cells.^44^ These cells maintain the antiviral cytokine and effector functions that are known to provide protection against viral reinfection in other viral pathogens.^39^ CD4+ memory T cells are polyfunctional, produce and secrete the cytokines interleukin 2, interferon γ, and tumour necrosis factor α, and are biased towards a follicular helper or type 1 helper phenotype.

**Figure 2:**
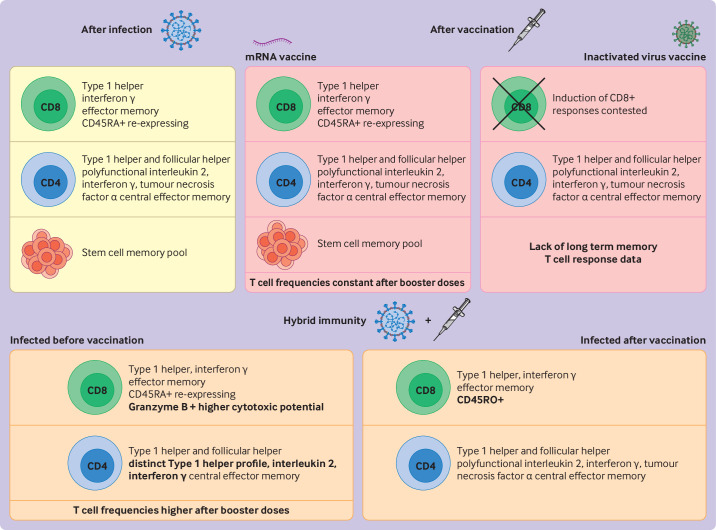
Summary of SARS-CoV-2 specific T cell memory phenotypes in different scenarios. Created with Biorender.com

In contrast with CD8+ T cells, CD4+ T cells show a primarily central memory effector (CCR7+ CD45RA−) phenotype eight months after infection which, with self-renewing capacity, indicates that SARS-CoV-2 specific T cell memory after infection might be long lasting and maintained for many years (figure 2). Memory T cell responses to the SARS-CoV-1 virus have been found in survivors after 17 years.^4^ The magnitude of memory T cell responses were similar in individuals who had or did not have symptoms of covid-19. Although the data are limited, memory T cells from individuals with no symptoms showed a proportional increase in interleukin 10 producing T cells compared with individuals with symptoms, and immunodominance towards accessory proteins. Memory T cells from individuals with no symptoms were not weak but highly functional.^45–47^ Functional memory T cells are also induced in the absence of active infection in close contacts of individuals with covid-19, although at a lower magnitude and with reduced polyfunctionality.^47^

Despite the spectrum of severity of covid-19 disease, no significant differences in the function or phenotype of immune memory responses have been found after SARS-CoV-2 infection in mild or severe disease. Memory T cell responses, including polyfunctionality and proliferative capacity, are maintained, regardless of the severity of covid-19 disease.^48 49^ Furthermore, no differences in the magnitude of memory responses have been recorded between men and women.^50^ Children rarely develop severe covid-19 disease, often having mild or asymptomatic disease. Children have a greater subset of stem cell memory T cells,^51^ and they quickly develop a robust T cell memory pool to SARS-CoV-2. Older adults, who are at higher risk of severe covid-19 disease and death, have impaired cytotoxic capacity,^39^ and severe covid-19 disease has been associated with lower T cell receptor diversity, lower T cell receptor avidity, and a reduced naive T cell repertoire.^52–55^

### T cell memory after covid-19 vaccination

Many of the currently approved covid-19 vaccines are highly immunogenic adenoviral (ChAdOx1) or mRNA based (BNT162b2) vaccines. These vaccines target the spike protein of the SARS-CoV-2 virus, a viral protein involved in cell entry and thus a logical vaccine target for attempting sterilising immunity. Systemic spike specific, SARS-CoV-2 specific T cells can be detected as early as seven days after the first dose of a covid-19 adenoviral or mRNA vaccine in individuals not previously infected with the virus, which parallels the kinetics of viral T cell specific induction seen in natural infection.^56^ Functional CD8+ T cells are mobilised earlier than CD4+ T cells, but a concerted CD4+ and CD8+ T cell response is seen.^57 58^ Encouragingly, robust stem cell memory T cell induction is found in most individuals, with both CD8+ and CD4+ stem cell memory T cells maintained for up to six months after vaccination in individuals not previously infected with the virus^59 60^ (figure 2). These multipotent memory cells, with high turnover, have been associated with long term CD8+ memory durability, assessed up to six months after vaccination, and might be a useful early indicator of effective memory induction.^60^

Similar to natural infection, long term vaccine induced spike specific, SARS-CoV-2 specific T cells have a CD45RO+ effector memory and CD45RA re-expressing effector memory phenotype, with follicular helper and type 1 helper polarisation also seen.^61 62^ After the first vaccine course, booster doses of mRNA vaccines have been shown to have little effect on spike specific CD8+ T cell memory frequencies, including the CD8+ stem cell memory pool, which remained constant after three and four vaccine booster doses,^63^ indicating a minimal effect of booster immunisation on long term CD8+ T cell memory.

Compared with adenoviral and mRNA based vaccines, humoral responses to inactivated virus vaccines (eg, CoronaVac and BBIBP-CorV) are less immunogenic, stimulating 10 times lower neutralising antibodies than mRNA vaccines. Waning of neutralising antibodies occurs as early as three months after vaccination, compared with mRNA vaccines where antibodies persist for up to six months. mRNA and adenoviral vaccines, however, currently only target spike antigen, whereas inactivated virus vaccines produce T cell responses from a wider breadth of SARS-CoV-2 antigens, including envelope and nucleocapsid antigens. Nucleocapsid antigen is much less prone to mutation than spike antigen. Although the magnitude of spike specific T cell responses is lower, the combination of the breadth of responses produced by inactivated virus vaccines is quantitatively comparable with mRNA vaccines.^34^

Inactivated virus vaccines induce a type 1 helper T cell response with a similar interferon γ and interleukin 2 secretion profile to mRNA vaccines, and a comparable spike immunodominance hierarchy. One observational cohort study of 126 participants, however, which robustly compared mRNA vaccines with inactivated vaccines, found that inactivated vaccines did not produce CD8+ T cell responses against any viral proteins.^34^ This finding is in contrast with other studies which showed induction of both CD4+ and CD8+ T cell responses by inactivated virus vaccines.^31 61 64^ This discrepancy might be because of differences in antigen specific T cell characterisation assays, highlighting the need for orthogonal assays to confirm true antigen specificity (figure 2).

CoronaVac and BBiBp-CorV have been used in almost half of the 7.3 billion doses of vaccine delivered to the world by the end of 2021,^65^ but a detailed analysis of the long term T cell memory response of inactivated vaccines compared with mRNA or adenoviral based vaccines is limited. This limitation is in part because of the bias in geographical location of the vaccine platform roll-out and the lack of research infrastructure for detailed T cell characterisation in some of the less wealthy nations (figure 2). Dealing with this bias will be important and ensuring that all vaccine platforms are considered equally in the changing landscape of the pandemic.

### T cell memory after infection or vaccination in individuals with compromised immune system

Individuals with immunodeficiencies represent a large clinically vulnerable group to SARS-CoV-2 infection. This group includes individuals with primary immunodeficiency who generally develop symptoms for a similar length of time as the general population, but in those with B cell pathway defects, including X linked agammaglobulinaemia, infections are often prolonged. In general, individuals with primary immunodeficiencies have a substantially higher rate of hospital admission (49%) after SARS-CoV-2 infection.^66 67^ T cell memory after covid-19 vaccination in individuals who are immunocompromised varies widely depending on the nature of the specific immunodeficiency or immunosuppression. For example, after mRNA vaccination, individuals with an acquired or inherited lack of mature B cells (eg, anti-CD20 treatment or X linked agammaglobulinaemia) developed a broad functional spectrum of CD4+ and CD8+ spike specific T cells, with CD8+ T cells expressing type 1 helper polarisation and an effector memory CD45RA+ and stem cell memory phenotype, similar to the general population, in the absence of humoral immunity, whereas CD4+ memory T cells were of lower magnitude six months after vaccination with a modest reduction in follicular helper T cells.^68 69^ Reflecting the diverse causes of immunodeficiency and immunosuppression, the functional quality of T cell memory is variable after vaccination.^69^ Individuals with solid organ transplants have particularly poor induction of T cell responses after vaccination, with lower magnitude, functionality, and durability.^69 70^

### Differences in T cell memory after infection or vaccination

One dose of the BNT162b2 mRNA vaccine induced a similar magnitude of spike specific T cell response as previous infection, measured in peripheral blood by interferon γ ELISpot.^71^ A substantial difference in SARS-CoV-2 T cell memory induced by infection or by the vaccine, however, was the ability to induce tissue mediated immunity. SARS-CoV-2 specific tissue resident memory T cells have mainly been seen after infection with the SARS-CoV-2 virus rather than after vaccination alone. Tissue resident memory T cells are defined as a subset of memory T cells that persist in epithelial barrier tissues, providing rapid in situ protective responses,^72 73^ and that can return to the wider circulation.^74^ Respiratory infections typically start at the respiratory epithelium, the site of encounter with the pathogen, and tissue resident memory T cells have been shown to correlate with protection against influenza virus^75^ and respiratory syncytial virus^76^ in murine infection models and in humans.

Efforts to explain the tissue resident memory T cell responses that follow infection with the SARS-CoV-2 virus have contributed to developing an understanding of the role of mucosal immunity in SARS-CoV-2 infection, and SARS-CoV-2 specific tissue resident memory T cells have been shown to be widely distributed in the bone marrow, spleen, lung, and multiple lymph nodes of seropositive donors.^77^ An early observational study of single cell RNA sequencing of cells in bronchoalveolar lavage fluid from 13 patients with acute covid-19 disease showed that patients with moderate disease were characterised by the presence of highly clonally expanded CD8+ T cells expressing tissue residence markers *ITGA1*, *CXCR6*, and *JAML*.^78^ Several studies have shown the persistence of tissue resident memory T cells in the lungs up to a year after acute SARS-CoV-2 infection.^77 79–81^ SARS-CoV-2 specific CD8+ T cells have also been shown to persist for at least two months after viral clearance in the nasal mucosa.^82^ Antigen specific tissue resident memory T cells are likely to have a role in mediating protective immunity to SARS-CoV-2 infection, and studies of T cell immunity after human challenge of individuals previously infected and not infected with the might be more informative.^83^

In comparison, SARS-CoV-2 specific tissue resident memory T cells were either absent^84^ in bronchoalveolar lavage fluid or limited in the lungs^85^ of individuals who were vaccinated with mRNA vaccines compared with those who were infected previously. In the upper respiratory tract, the ability of mRNA vaccines to produce antigen specific functional tissue resident memory T cells is still unclear, owing partly to technical limitations in sampling nasal washes or nasal swabs.^84^ Although some studies have reported an expansion of tissue resident memory T cells in the nasal mucosa after vaccination with mRNA vaccines,^86 87^ others have argued that SARS-CoV-2 specific tissue resident memory T cells in the nasal mucosa can only be detected after breakthrough infection.^88^

### Hybrid immunity, influence of variants, and population level immunity

Hybrid immunity to the SARS-CoV-2 virus is defined as immunity created by a combination of vaccination and infection. This type of immunity can include different infection statuses before vaccination, from the SARS-CoV-2 virus and from seasonal coronaviruses, different vaccine regimens and boosters, and infection after vaccination caused by the omicron variant of the SARS-CoV-2 virus and sublineages. Hybrid immunity creates the most robust immunity against SARS-CoV-2 variants, with broad responses to multiple SARS-CoV-2 proteins identified systemically and at mucosal sites.^29 30 71 89^ A recent large systematic review of 26 studies showed that hybrid immunity generated the most effective and durable protection against reinfection, hospital admission, or severe disease, remaining >95% over 11 months of follow-up.^90^

As the pandemic has progressed, reinfection with the omicron variant of the virus and sublineages after vaccination has become relatively common, with most of the population having hybrid immunity status. Understanding the role of T cell memory in hybrid immunity, and the response to emerging variants, is important to understand how we can maintain immunity and protection at the population and individual level.

T cell memory in hybrid immunity was assessed after three doses of a mRNA vaccine (mRNA-1273 or BNT162b2) over about eight months, showing that SARS-CoV-2 infection before vaccination produced a distinct population of type 1 helper CD4+ memory T cells expressing interferon γ and interleukin 10. This response was not reproduced in individuals not infected with the virus who received three doses of a vaccine, suggesting that previous infection imprints the memory response.^91^ An observational cohort study of 684 participants receiving two doses of ChAdOx1-S or BNT162b2 followed by a third dose of BNT162b2 showed that T cells were boosted by the third dose, were well maintained for at least six months after the booster dose, and that previous infection continued to give a higher magnitude of T cells even after three vaccinations^29^ (figure 2) .

Although no significant difference was seen in the phenotype of memory CD8+ T cell responses in individuals previously infected and then who were vaccinated compared with individuals who were not previously infected and then who were vaccinated,^92 93^ this response was characterised by a predominance of CD8+ effector memory T cells re-expressing CD45RA. This CD8+ T cell memory phenotype was lacking in individuals with hybrid immunity characterised by infection after vaccination, who showed a predominantly CD8+ T cell effector memory phenotype. The spike specific CD8+ effector memory cells re-expressing CD45RA, identified in individuals with hybrid immunity (ie, those who were infected and then received a vaccine), were found to express more granzyme B mRNA, suggesting potentially greater cytotoxic potential.

As new variants have emerged, many groups have studied whether T cell memory induced by hybrid immunity, particularly from infection before vaccination, shows functional reactivity to these variants. In individuals previously not infected with the virus, multiple studies have shown that T cell memory induced by different vaccines (mRNA-1273, BNT162b2, Ad26.COV2.S, and NVX-CoV2373) showed spike specific CD8+ and CD4+ T cell responses with cross reactivity against variants, including the delta and omicron variants of the virus,^29 30 92 94–96^ with 90% (CD4+) and 87% (CD8+) of memory T cell responses preserved against variants, on average, assessed by the activation induced marker assay.^35^

In contrast, an observational study of 731 participants that used interferon γ ELISpot suggested that previous infection suppresses the ability of T cells to recognise mutated regions of omicron spike protein.^97^ Although this finding is concerning because it indicates viral escape, hybrid T cell immunity has been shown to produce a broad T cell response to multiple proteins, not just to spike antigen, and the role of non-spike SARS-CoV-2 antigens was not considered.^97^ A comparable observational cohort study of 94 participants, which also used interferon γ ELISpot, showed that non-spike responses increased substantially after infection with the omicron variant of the virus in individuals who had received three doses of vaccine, regardless of previous infection status.^98^ Furthermore, an observatioanl study of 40 participants that assessed CD4+ and CD8+ T cell responses to spike antigen by intracellular cytokine staining in individuals who had been vaccinated, showed that polyfunctionality of memory T cell responses to the omicron variant of the virus was preserved in both individuals previously infected with the virus and in those not infected, with only modest differences in the frequency of responses seen.^29 99^

Assessment of T cells by MHC multimers and scRNAseq to profile antigen specific T cells from multiple SARS-CoV-2 proteins after repeated exposure to antigens, including vaccination, previous infection, and breakthrough infection, found no evidence of narrowing of the T cell repertoire from repeated exposure.^93^ Repeated exposure, including those with omicron breakthrough infection after spike based vaccination, produced a broad CD8+ T cell response that might better prepare individuals against future new variants.

Hybrid immunity also includes individuals with cross reactive T cell responses to seasonal coronaviruses. The presence of T cell cross reactivity to human coronaviruses, which can be identified in about 50% of individuals, was established quickly at the beginning of the pandemic, although the significance of these responses and their role in the development of memory T cell responses to SARS-CoV-2 infection and vaccination has yet to be fully defined.^4 44 100–103^ Cross reactive human coronavirus specific T cells are mostly absent from the T cell repertoire in older people but are seen in younger adults and children.^104–106^ Patients with pre-existing cross reactive CD4+ T cell memory have been suggested to have a stronger CD4+ T cell response to the vaccine (ie, of higher magnitude, more polyfunctional and a greater proportion of follicular helper T cells).^62^ An intact naive repertoire and that exclusion of pre-existing memory T cells is required for effective expansion of spike specific T cells against the SARS-CoV-2 virus after spike based vaccination (ChAdOx1-S and BNT162b2)^107^ has been argued, however, and also that human coronavirus specific T cells are often of low avidity against SARS-CoV-2 peptides.^52 108^

### Correlates of protection

#### Role of T cells in immune protection

**Figure 3:**
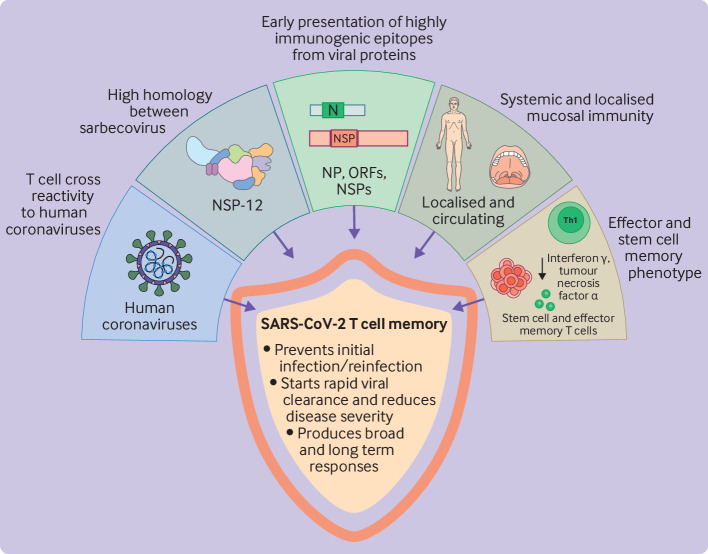
Key features of SARS-CoV-2 specific T cell memory correlating with protection, summarising the role of SARS-CoV-2 T cell memory as well as the potential underlying mechanisms. NSP=non-structural protein; NP=nucleocapsid protein; ORF=open reading frame; Th1=type 1 helper T cell. Created with BioRender.com

Systemic SARS-CoV-2 specific T cells can be detected in patients who have recovered from covid-19 disease, as well as in individuals with no symptoms, in individuals who were vaccinated, or in individuals exposed to the virus but who did not seroconvert.^105 109–111^ Given the complex interplay between various components of the adaptive and innate immune system, determining the precise role of T cell memory in clinical protection is challenging (figure 3). Accumulating evidence suggests that T cell memory is important in helping to clear infection and reduce viral loads, as might be expected, but it can also prevent the first infection or reinfection.

Although a correlation between the presence of SARS-CoV-2 specific T cells and protection from severe disease was initially unclear,^55 112 113^ numerous longitudinal patient cohort studies have shown that a coordinated adaptive response with rapid expansion of both T cells and circulating antibodies, within seven days of infection, correlated with protection from severe disease.^53 114–116^ Whereas a delayed adaptive response seems to be correlated with early inflammation and more severe covid-19 disease,^117 118^ with patients often having severe lymphopenia,^119 120^ this phenomena is not uncommon in other infections. Also, evidence of SARS-CoV-2 specific T cell memory in individuals exposed but not infected, so-called abortive infection, suggests that T cells have a defining role in rapid viral clearance and protection.^105 110^ These correlative studies have been confirmed by mechanistic studies in animal models, including non-human primate models that supported the role of CD8+ T cells, particularly in the case of waning antibody protection,^121^ and in phase 1 trials of adoptive T cell treatment with SARS-CoV-2 specific CD45RA memory T cells from convalescent donors to treat severe covid-19.^122 123^

#### Magnitude, breadth, durability, and function in T cell mediated protection

To what extent the magnitude of the memory T cell response alone, after infection or vaccination, protects against infection or severe disease is unclear, with studies indicating that coordinated humoral and cellular high responses correlated with protection against breakthrough infection.^124–127^ T cell magnitude correlates with the level of protein expression from the corresponding SARS-CoV-2 gene.^101 116^ The breadth, function, and durability of SARS-CoV-2 memory T cells induced by the vaccine or by infection have an important role in real world effectiveness. As discussed previously, the different vaccines induce a different breadth of response, most notably with mRNA vaccines (BNT162b2 and mRNA-1273) only inducing spike specific CD4+ and CD8+ T cell responses^92^ whereas infection induces CD4+ and CD8+ T cell responses to structural and non-structural proteins across the whole SARS-CoV-2 genome.^55 101 115^

In contrast, where this response has been assessed, inactivated virus vaccines induced CD4+ (but not CD8+) T cell responses against spike, nucleocapsid, and membrane proteins.^34 128^ Overall phenotype and magnitude were found to be comparable for all vaccines, but vaccine efficacy against infection, hospital admission, and death was high (<90%), irrespective of breadth. Whether an increased breadth of response against non-spike antigens offers more long term protection against emerging variants of concerns is unclear. Effective control and protection from less severe disease has been associated with SARS-CoV-2 specific CD4+ type 1 and follicular helper T cell responses,^53^ and a coordinated CD8+ type 1 effector response, whereas the role of other CD4+ helper subsets (type 2, type 17) are unclear.^120 129–131^ Memory responses after infection and vaccination are characterised by an effector memory CD45RA+ response, although this memory phenotype was largely absent in individuals who were infected after vaccination.^93^

Furthermore, a substantial population of SARS-CoV-2 specific memory CD8+ T cells are characterised by a stem cell memory phenotype, which with self-renewing capacity has the potential to support the memory pool for many years after infection or vaccination. T cell memory is substantially more durable than humoral immunity, particularly for emerging viral variants, where neutralising antibodies have been found to wane considerably.^30 132^ Durable T cell memory induced by infection or vaccination has been suggested to be protective against severe disease for more than 14 months, irrespective of variant, whereas protection against reinfection wanes. Some modest protection still exists against all variants, however, including the omicron variant, up to a year after the first infection.^133^ Although most of these studies offer only causal links, given the durability, breadth, and functional phenotype of the T cell memory response, speculating that T cell memory has a major role in contributing to this ongoing protection is tempting.

#### Immunodominance, cross reactivity, and hybrid immunity in T cell mediated protection

Evidence for a clear hierarchy around epitope targeting and immune protection after SARS-CoV-2 infection is still emerging,^43 134–136^ and immunodominance does not necessarily translate to immune protection. Rather than immunodominance, immunogenicity should be considered (ie, how quickly and robustly a viral protein can produce an effective T cell response).

T cells that can recognise viral proteins presented by MHC at an early stage of viral infection, before de novo production of virions, might have a protective advantage in SARS-CoV-2 infection by limiting viral spread or even by causing an abortive infection. This situation is suggested to explain the expansion of non-structural protein 12 specific T cells in individuals who were highly exposed to the SARS-CoV-2 virus but who had a negative PCR test result.^110^ Moreover, viruses can rapidly suppress MHC expression on the cell surface after infection and therefore early presentation of epitopes might be critical for controlling viral spread. This theory is supported by a SARS-CoV-2 proteomic analysis, where early expressed viral proteins dominated HLA-I presentation and immunogenicity.^137^ These immunogenic viral proteins included non-structural proteins and out-of-frame open reading frames of the SARS-CoV-2 virus, which despite their low abundance in infected cells generated multiple epitopes for HLA-I presentation in the first 6-12 hours of infection, and highly immunogenic responses (figure 3). In previous studies that aimed to identify correlates of immune protection in HIV, early presentation of highly immunogenic epitopes was proposed to underpin the protective mechanism of CD8+ T cell responses in individuals infected with HIV with long term non-progression to AIDS, in which infected target cells could be sensitised for lysis within six hours of infection.^138–140^

In covid-19 disease, evidence from animal studies indicates that nucleocapsid T cell responses are associated with less severe disease and lower viral loads,^141^ and in humans, nucleocapsid induces some of the strongest CD8+ T cell responses in natural infection, with multifunctional nucleocapsid specific CD8+ T cells associated with mild disease.^55^ In support of nucleocapsid as an effective antigenic target, HLA-B*0702 NP105-113 has been shown to produce strong antiviral immunity and protection against severe disease.^142^ Furthermore, a mRNA vaccine (BNT162b4) containing immunogenic regions of SARS-CoV-2 nucleocapsid, membrane, and open reading frame 1ab, protected against severe disease and reduced viral titres on challenge with viral variants in animal models, producing diverse polyfunctional CD4+ and CD8+ T cell responses while maintaining the spike specific responses when co-administered with BNT162b2.^143^

The role of cross reactive memory T cells after SARS-CoV-2 infection and their relevance to protection is contentious, with multiple studies showing conflicting findings. Cross reactive memory T cells in a substantial proportion of individuals before exposure to the SARS-CoV-2 virus has been firmly established.^54 101–103^ The role of cross reactive memory T cells in protection is unclear, however, with some studies linking the presence of low avidity cross reactive CD4+ T cells clones with the severity of covid-19 disease^4 52 100 102 108^ and others indicating that these cells could facilitate expansion of effective T cell memory to SARS-CoV-2 after vaccination or infection, or both.^62 136 144 145^

Further evidence of a protective role of cross reactive SARS-CoV-2 memory T cells is the finding that recent infection with human coronaviruses can be linked to the development of less severe covid-19 disease.^146^ Also, a range of cross reactive epitope specific T cells was shown to expand in individuals exposed to the SARS-CoV-2 virus who never had a positive test result by PCR, suggesting these cross reactive memory T cells were highly effective at controlling viral infection.^105 110^ Cross reactive T cell memory to non-structural protein 12 (RNA dependent RNA polymerase) SARS-CoV-2 protein, which was detected in healthy individuals and in those highly exposed to the virus, might be of interest because of the high level of homology in this protein between different sarbecovirus. A recent cohort study of 88 participants reported common immunodominant regions conserved across coronaviruses, which could help in developing a pan-coronavirus response and in protecting against future pandemic strains.^147^

SARS-CoV-2 memory T cells cross reactive to human coronaviruses are largely absent in the T cell repertoire of older adults, a clinically vulnerable group with an increased risk of severe covid-19 disease,^106^ but these T cells are seen in young children.^148^ Although decreasing thymic activity reduces new naive T cells in elderly people, results from next generation sequencing of T cell receptor libraries challenged the paradigm that elderly people have a reduced naive T cell repertoire. Elderly people had a diverse naive T cell repertoire but a highly abnormal clonal expansion of some naive T cell populations, with increased inequality in clonal size correlating with age.^149^ This change in the naive T cell pool could severely compromise epitope responsiveness. The composition of the antigen specific pre-exposure T cell repertoire is a key determinant of the quality of the SARS-CoV-2 immune response to vaccination, requiring both a diverse naive compartment and some cross reactive memory T cells; in elderly people, T cell expansion from both compartments is severely compromised.^107^ Hybrid immunity created by vaccination and infection has been shown to produce broad T cell memory to multiple SARS-CoV-2 proteins, both systemically and at mucosal sites, and to provide better T cell mediated protection from variants, with more rapid control of virus replication, than individuals who received three doses of vaccine alone.^150^

On balance, these data provide evidence to support the inclusion of non-structural proteins, such as the highly conserved and immunogenic non-structural protein 12, nucleocapsid, and out-of-frame open reading frame in the design of next generation vaccines, especially given the spike centric vaccines offered by some of the leading vaccine platforms. Furthermore, targeting highly conserved immunogenic regions in future vaccines might produce pan-coronavirus vaccine responses, helping to future proof against coronavirus infection. Future vaccine designs must consider tissue accessibility to promote tissue resident memory T cell mediated protective function in the respiratory tract against the SARS-CoV-2 virus. Vaccination in elderly people, however, might require different strategies to produce high quality T cell responses against new pathogens.

## Emerging treatments

The respiratory tract is the site of viral entry and transmission in the body, and therefore mucosal booster vaccines that produce robust mucosal memory T cell responses might be more effective at preventing transmission and reinfection and reducing disease burden. Producing robust mucosal immunity while maintaining protection against severe disease is a priority for next generation covid-19 vaccines. At least 44 mucosal vaccines are in preclinical development, although none has been approved for use by regulatory agencies in the US or Europe.^151^ The robust protective immunity driven by a hybrid immune status might in part be a result of diverse immunogenic regions targeted by T cell responses, including non-structural proteins such as non-structural protein 12, nucleocapsid, and out-of-frame open reading frame. After success in animal models, BNT162b4, which produces T cell responses against diverse non-spike epitopes, is currently being clinically evaluated in combination with the BA.4/BA.5 39 omicron updated bivalent BNT162b2 (Safety and Effects of an Investigational Covid-19 Vaccine as Booster in Healthy People, NCT05541861).

## Conclusions

Multiple concurrent studies of SARS-CoV-2 infection and vaccination have indicated that T cell memory has a key role in preventing severe disease and limiting reinfection. Evidence indicates that T cell memory might provide rapid protection through cross reactivity to human coronaviruses, targeting of early viral epitopes for viral clearance, and prevention of infection without seroconversion, and recognise viral variants mediating the effects of viral escape. Most of these studies were based on easily accessible peripheral blood in observational human cohorts, but now more detailed mechanistic studies will be required to confirm the correlates of protection identified. More detailed mechanistic studies will also help inform future vaccine strategies, particularly the role of mucosal immune memory in protection and the benefit of non-structural proteins as vaccine targets.

**Questions for future research**

- What is the role of mucosal T cell memory for effective prevention of disease?
- How does cross reactivity between coronaviruses influence T cell memory and protection in the long term and against new strains?
- How does vaccination induce protection against any infection versus severe disease, and how long does this protection last?

**Patient involvement**

Patients and/or the public were not involved in the design, or conduct, or reporting, or dissemination plans of this research.
